# SJS/TEN following therapy with pembrolizumab and enfortumab vedotin in a patient with urothelial carcinoma

**DOI:** 10.1016/j.jdcr.2026.02.023

**Published:** 2026-02-17

**Authors:** Natalie Braun, Vrinda Madan, Sepideh Ashrafzadeh, Jordan Said, Vinod E. Nambudiri

**Affiliations:** a Department of Dermatology, Brigham and Women’s Hospital, Boston, Massachusetts; b Harvard Medical School, Boston, Massachusetts; c Department of Dermatology, Johns Hopkins University School of Medicine, Baltimore, Maryland

**Keywords:** cutaneous drug toxicity, enfortumab vedotin, nectin-4, SJS/TEN, Stevens-Johnson syndrome, toxic epidermal necrolysis, urothelial carcinoma

A 59-year-old male with metastatic urothelial carcinoma developed pruritic patches on his thighs 5 days after initiating pembrolizumab and enfortumab vedotin (EV). His oncologist prescribed triamcinolone 0.1% ointment, and he was given a second dose of EV without pembrolizumab. Four days later, he presented to the emergency department for persistent erythematous patches with worsened pruritus on the trunk and extremities (Fig 1). Clobetasol 0.05% cream was prescribed and pembrolizumab and EV were held. Two days later, he returned to the emergency department with widespread erosions and Nikolsky positive bullae (Fig 2). There was no involvement of oral, ocular, or anogenital mucosae. The patient was admitted and given 1.0 mg/kg/day methylprednisolone for 3 days and subsequently transitioned to 60 mg prednisone for 3 days and then tapered. Imaging 1 month after discharge showed excellent oncologic response, and no additional therapy was initiated.

**Fig 1:**
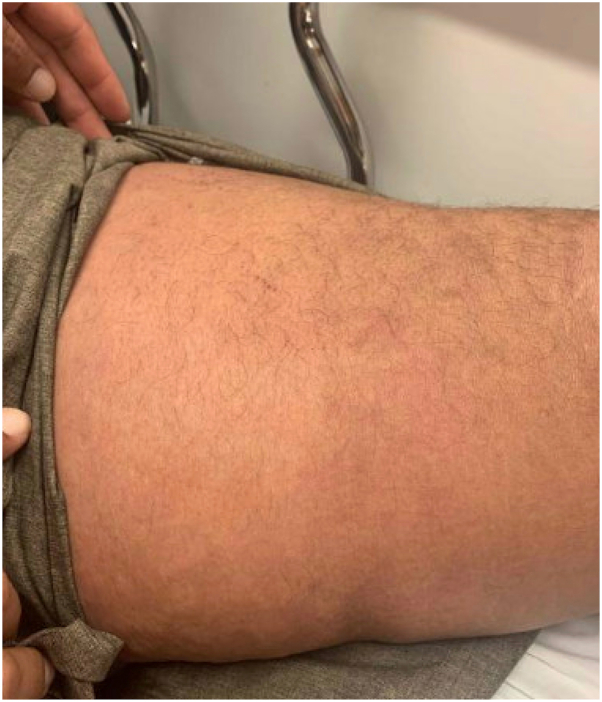
Initial emergency department presentation with erythematous patches.

**Fig 2:**
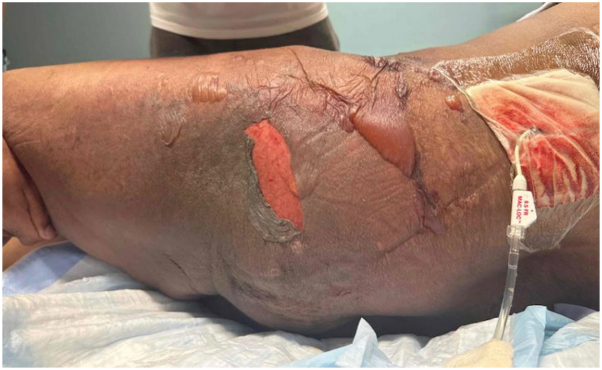
Representation to the emergency department 2 days later with widespread superficial erosions and flaccid bullae.

**EV targets Nectin-4. What is the role of Nectin-4 in the skin?**

**A.** Regulation of sebaceous glands
**B.** Maintenance of epithelial tissue integrity through cell adhesion
**C.** Modulation of melanin production
**D.** Promotion of keratinocyte differentiation
**E.** Blockade of T-cell programmed death receptor-1

The correct answer is B: Maintenance of epithelial tissue integrity through cell adhesion. EV is an antibody-drug conjugate that targets Nectin-4, a cell adhesion protein that induces cell apoptosis by disrupting microtubule function.^1^ Nectin-4 is highly expressed on both urothelial cancer cells and keratinocytes. Stevens-Johnson Syndrome/Toxic Epidermal Necrolysis (SJS/TEN) is a life-threatening adverse drug reaction where the immune system targets keratinocytes, leading to full thickness epidermal necrosis. EV is associated with cutaneous adverse effects,^2^ and there have been reports of EV-associated skin reactions that present as SJS/TEN (EV-associated SJS/TEN).^3^ Inhibition of Nectin-4 in the skin may contribute to the higher risk of SJS/TEN with this medication.^1^^,^^3^ The US Food and Drug Administration has updated prescribing information for EV to include SJS/TEN as an adverse event.

Both classic and EV-associated SJS-TEN share clinical features such as widespread Nikolsky positive bullae and mucocutaneous erosions. However, important distinctions exist between these reactions. Classic SJS/TEN is a delayed (type IV) hypersensitivity reaction mediated by drug-specific CD8+ T-cells. In contrast, EV-associated SJS/TEN involves both immune-mediated mechanisms and direct Nectin-4-mediated keratinocyte toxicity. Additionally, patients receiving EV may present with clinical features suggestive of SJS/TEN, but histopathology can reveal findings consistent with toxic erythema of chemotherapy (TEC).^4^ EV exerts cytotoxicity via the release of the microtubule inhibitor monomethyl auristatin E, leading to keratinocyte cytotoxicity and the potential development of TEC. Biopsy is therefore essential, as distinguishing between TEC and SJS/TEN is necessary to determine appropriate treatment. This patient did not develop mucosal involvement. Cases of EV-associated SJS/TEN without mucosal involvement have been reported, in contrast to classic SJS/TEN where mucosal involvement occurs in 90% to 100% of cases.^5^ Additionally, the early-onset, intertriginous distribution suggested EV-induced toxicity over a pembrolizumab-associated immune-related adverse event.

We present a case of SJS/TEN likely secondary to EV with rapid progression from a benign-appearing rash to widespread erosions within 2 days, highlighting the need for vigilant monitoring even in early or mild presentations.

## Conflicts of interest

None disclosed.
